# Bactericidal and Anti-Biofilm Activity of Ethanol Extracts Derived from Selected Medicinal Plants against *Streptococcus pyogenes*

**DOI:** 10.3390/molecules24061165

**Published:** 2019-03-24

**Authors:** Niluni M. Wijesundara, H. P. Vasantha Rupasinghe

**Affiliations:** 1 Department of Biology, Faculty of Science, Dalhousie University, Halifax, NS B3H 4R2, Canada; niluniw@dal.ca; 2 Department of Animal Science, Faculty of Animal Science and Export Agriculture, Uva Wellassa University, Badulla 90 000, Sri Lanka; 3 Department of Plant, Food, and Environmental Sciences, Faculty of Agriculture, Dalhousie University, Truro, NS B2N 5E3, Canada; 4 Department of Pathology, Faculty of Medicine, Dalhousie University, Halifax, NS B3H 4R2, Canada

**Keywords:** streptococcal pharyngitis, antibacterial agents, phytochemicals, natural health products, *Streptococcus pyogenes*, infectious disease

## Abstract

**Background:** There is a growing interest in medicinal plants which have been traditionally used for the treatment of human infections. This study assessed 14 ethanol extracts (EEs) on bacterial growth and biofilm formation of *Streptococcus pyogenes*. **Methods:** Constituent major phytochemicals in the extracts were identified using ultra performance liquid chromatography-electrospray ionization-tandem mass spectrometry (UPLC-ESI-MS/MS). Micro-broth dilution and time-kill assays were used to determine antibacterial activities. Anti-biofilm activities were studied using MTT assay, and morphology of biofilms was observed by scanning electron microscopy (SEM). Transmission electron microscopy (TEM) was employed to visualize the ultra-cross section structure of bacteria treated with efficacious extracts. **Results:** Licorice root, purple coneflower flower, purple coneflower stem, sage leaves and slippery elm inner bark EEs were the most effective, with minimum inhibitory concentrations (MIC) and minimum bactericidal concentrations (MBC) of 62.5 μg/mL and 125 μg/mL, respectively. The minimum biofilm inhibitory concentration (MBIC) of extracts ranged from 31.5–250 μg/mL. Morphological changes were observed in treated biofilms compared to the untreated. The four most effective extracts exhibited the ability to induce degradation of bacterial cell wall and disintegration of the plasma membrane. **Conclusion:** We suggest that EEs of sage leaf and purple coneflower flower are promising candidates to be further investigated for developing alternative natural therapies for the management of streptococcal pharyngitis.

## 1. Introduction

*Streptococcus pyogenes*, also called group A streptococcus (GAS), is one of the most important a Gram-positive cocci. It is the major bacterial cause of pharyngitis which is responsible for about 600 million cases of streptococcal pharyngitis on an annual basis worldwide [1]. Streptococcal pharyngitis requires appropriate antibiotic therapy [2] to prevent the transmission of the infections and to prevent superlative and non-superlative complications associated with streptococcal pharyngitis [3]. Although first-generation cephalosporin (cephalexin), macrolides (azithromycin), or clindamycin are also effective against GAS, penicillin is considered as the first choice of drug for many cases [3,4], where its resistance to GAS has never been documented. However, there are several treatment challenges, such as poor patient compliance, patient allergies, and unnecessary side effects having been reported [3]. The symptoms of streptococcal pharyngitis could overlap with viral pharyngitis; consequently, it is important to confirm GAS pharyngitis by proper diagnosis test before prescribing antibiotic therapy. Over-prescribing of antibiotics for pharyngitis is a growing concern that could lead to antibiotic resistance [5,6].

The interest in using plant extracts for the treatment of streptococcal pharyngitis has increased due to several treatment challenges associated with conventional antibiotics. Herbal plants are a great source of alternative treatments for many infections. Plants are rich in a wide variety of secondary metabolites (phytochemicals), such as polyphenols, flavonoids, terpenoids, alkaloids, and tannins [7], which have been shown to possess antimicrobial properties [8,9,10,11]. Thyme, oregano, sage, barberry, purple coneflower, and licorice are common medicinal plants used in traditional or folk healing practices in Canada to treat bacterial infections, including streptococcal pharyngitis. However, their potential activities on growth inhibition and biofilm formation of *S. pyogenes* by phytochemical-rich ethanol extracts of these plants have not been reported. Therefore, the current investigation was carried out to identify the most efficient plant source/s from twelve different herbal plants. 

The specific objectives were to identify the most active plant extracts against planktonic *S. pyogenes*, investigate the time required to kill bacteria, visualized treated bacteria cell wall and membrane damage, explore the inhibitory effects of efficacious extracts on biofilm formation inhibition, and visualize bacterial biofilm at their minimum sub-inhibitory biofilm concentrations.

## 2. Results

### 2.1. Characterization of Chemical Constituents Using UPLC-ESI-MS/MS

**Figure 1:**
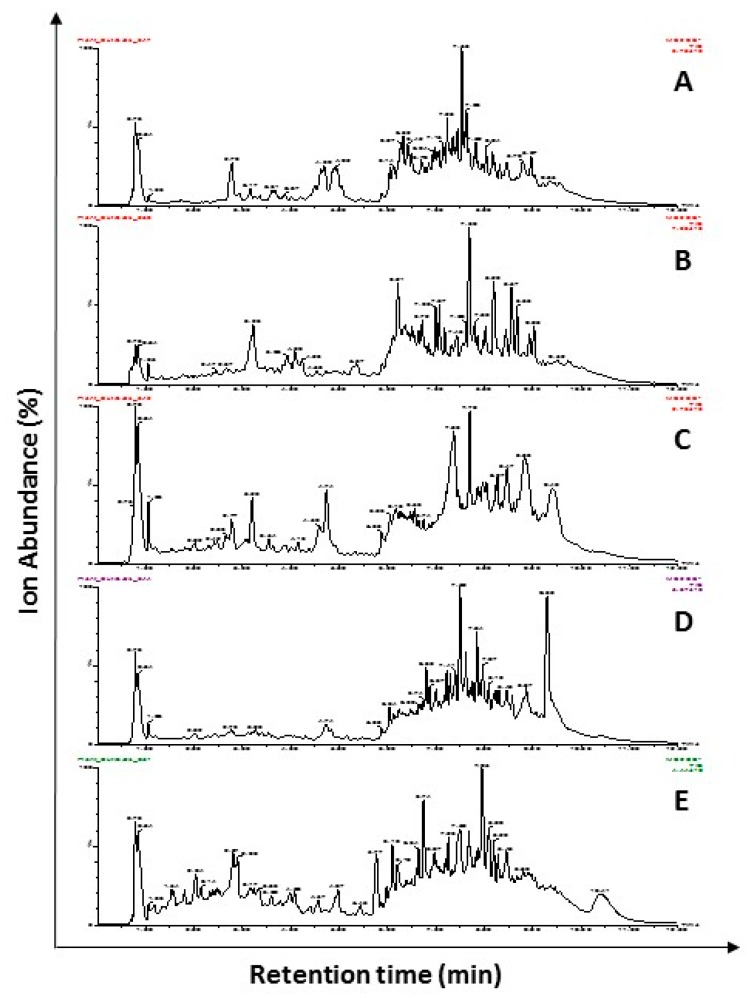
Total ion chromatograms (TIC) of deprotonated selected ion monitoring modes spectra of UPLC-ESI-MS/MS analysis of ethanol extracts of licorice roots (**A**), sage leaves (**B**), purple coneflower flowers (**C**), purple coneflower stems (**D**), and slippery elm inner barks (**E**). Full scan followed by SIM mode of UPLC-ESI-MS/MS was performed for the identification of phytochemicals present in the extracts. Relative ion absorbance vs. retention times is shown in TIC.

The deprotonated molecular mass ([M − H^+^]^−^) and retention time (RT) of each identified compound of all extracts using ultra-performance liquid chromatographic-electrospray ionization-tandem mass spectrophotometry (UPLC-ESI-MS/MS) are summarized in Table 1. The total ion chromatograms (TIC) of the most effective five extracts are shown in Figure 1. The identified constituents of each extract using full scan and selected ion monitoring (SIM) modes were verified by previously reported deprotonated ions in the same plant or other plant materials. An example of a full scan spectra and SIM mode chromatograms for sage EE is presented in Figure S1.

### 2.2. Inhibitory Effects of Plant Extracts against Planktonic S. pyogenes Growth

The antibacterial activities of 14 ethanol extracts (from twelve medicinal plants used in Canadian traditional medicine for streptococcal infections) were evaluated against two American Type Culture Collection (ATCC) *S. pyogenes* strains (ATCC 19615, ATCC 49399) and a clinical isolate from a pharyngitis patient. Penicillin G was used as the positive control. The minimum inhibitory concentration (MIC) and minimum bactericidal concentration (MBC) values are presented in Table 2. Out of these total 14 extracts, 10 possessed various levels of antibacterial effect against *S. pyogenes* within the tested range. The MIC and MBC values of the rest of the extracts were beyond the highest concentration of the test. Licorice root, sage leaves, slippery elm inner bark, purple coneflower flower, and stem EEs showed the highest inhibitory effects, with MIC values of 62.5 µg/mL, and shared a similar MBC value of 125 µg/mL for all bacterial strains (Table 2). 

### 2.3. Time to Kill S. pyogenes 

**Figure 2:**
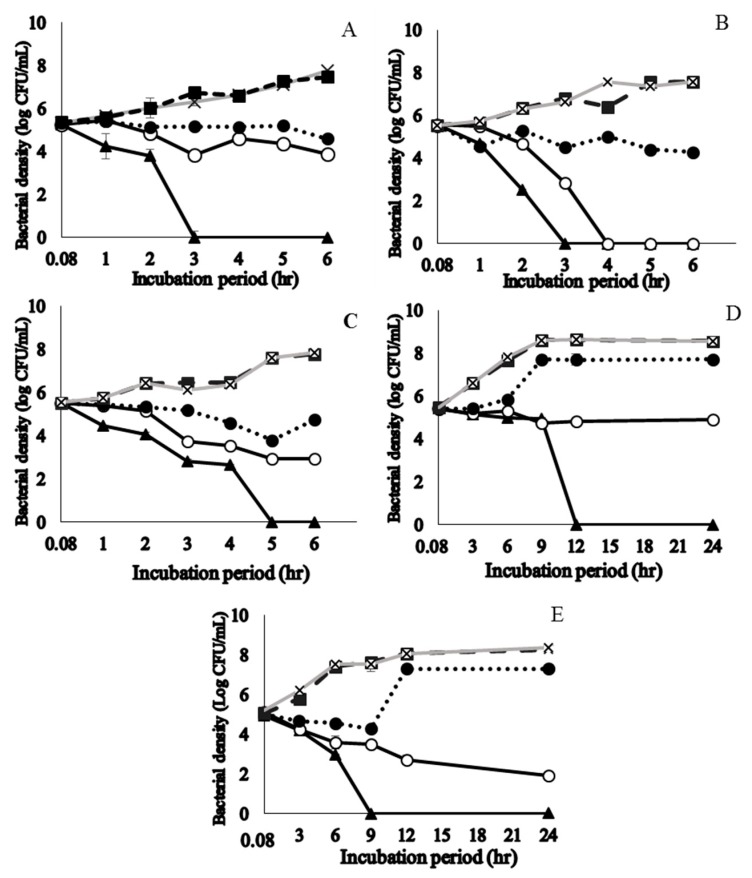
Time to kill curves for ethanol extracts of licorice roots (**A**), sage leaves (**B**), purple coneflower flowers (**C**), purple coneflower stems (**D**), and slippery elm inner barks (**E**) on the growth of *Streptococcus pyogenes* ATCC 19615. A viable count was performed for different concentrations at 0.08, 1, 2, 3, 4, 5, and 6 h incubation time points for A, B, C. Incubation periods of 0.08, 3, 6, 9, 12, 18, and 24 h were used for D and E. The killing curve was prepared at 35 ± 2 °C incubation in duplicate and the results were identical within each dilution. ▲ = 2 × MIC; ○ = MIC; ● = ½ × MIC; ■ = Diluent control (1% ethanol); × = bacteria control; MIC: minimum inhibitory concentration.

Time taken to achieve 99.99% planktonic *S. pyogenes* kill with the phytochemical-rich extracts were assessed. Licorice root, sage leaf, purple coneflower stem, purple coneflower flower, and slippery elm inner bark extracts were selected based on their significantly low MIC and MBC values. Time to kill assay results of those plant extracts showed a concentration and time-dependent action (Figure 2). Licorice root and sage extracts presented a bacteriostatic effect for the first 2 h of exposure; from this time on, they established a progressive decrease in bacteria cell count and showed a bactericidal effect after 3 h. Purple coneflower flower extract provided a bacteriostatic effect for the first 4 h of exposure, after which, it established its bactericidal effect. More than 99.9% of the initial inoculum of the strain of S. pyogenes was killed after 9 and 12 h exposure to concentrations of their respective 2 × MIC concentrations of slippery elm EE, purple coneflower stem EE. At their respective MIC value, purple coneflower flower EE and slippery elm EE rapidly became bactericidal against S. pyogenes, producing a more than three log reduction in viable counts within 24 h. No regrowth was observed by 24 h incubation for above five EEs. There was no significant growth inhibition in cells treated with 0.1% ethanol, which does not show a significant difference in bacterial density along with brain heart infusion (BHI) media control.

### 2.4. Transmission Electron Microscopy (TEM) Visualization of Fixed Biofilms of S. pyogenes

**Figure 3:**
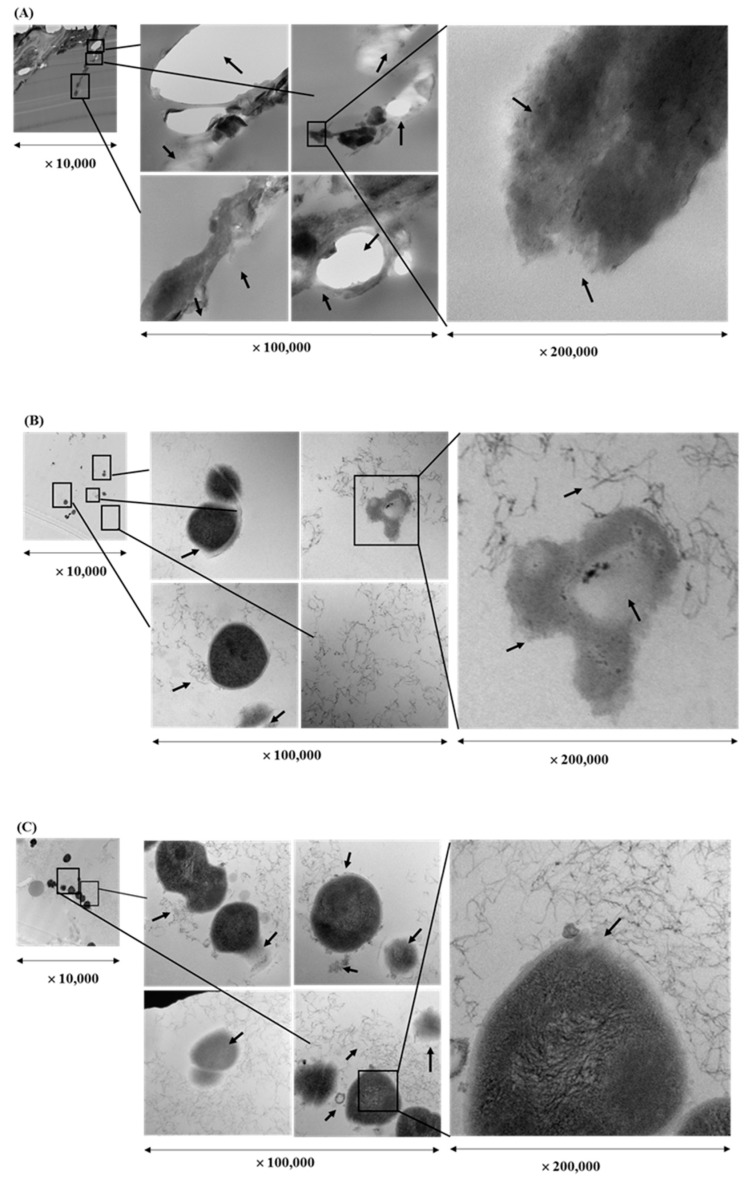

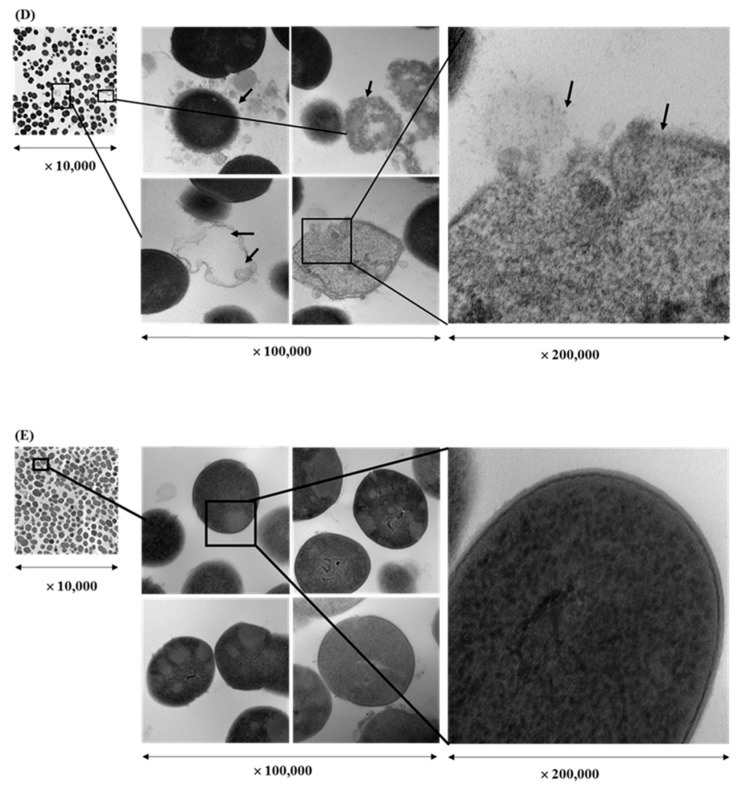
Transmission electron microscope image of a cross-section through *Streptococcus pyogenes* treated with ethanol extract of different herbs at a sub-inhibitory concentration (1/2 × MIC) for 16 hr: Licorice root (**A**), sage leaf (**B**), purple coneflower flowers (**C**), purple coneflower stems (**D**) extracts, and untreated control (**E**).

Cell morphology of bacteria treated with EEs of licorice roots, sage leaves, purple coneflower flowers, and purple coneflower stems was compared with intact bacterial cells in the untreated control (Figure 3). Untreated cells remained cocci shaped, regular, intact, and presented the distinctive characteristics of the striated cell wall and discernible cell membrane. It was also clearly shown electron-dense material inside the intact cells in dark color whereas small amounts of condensed substances were observed in the *S. pyogenes* cells treated with EEs. Comparatively, in the cells treated with each EE treatments at their sub-inhibitory concentrations showed deformed and shriveled morphologies in cell envelop and distracted membrane integrity. Furthermore, due to the massive leakage of intracellular contents, debris of either or both cell walls and cell membranes were observed in the TEM images of EE-treated bacterial cells. Cell densities compared to untreated control were found significantly low in all treated samples.

### 2.5. Anti-Biofilm Formation Activity

The effects of sub-inhibitory concentrations of the five efficacious extracts (licorice root, sage leaves, purple coneflower flower, purple coneflower stem, and slippery elm inner bark EEs) based on the MICs and time to kill curve results were further evaluated for their ability on the biofilm formation. Viable cells in the biofilms of the three *S. pyogenes* strains were quantified which treated with EEs over 72 h incubation using 3-(4,5-dimethylthiazol-2-yl)-2,5-diphenyltetrazolium bromide (MTT) staining. The MICs of five extracts and penicillin G were shown in Table 3. Penicillin G possessed a pronounced biofilm inhibition with significantly lower minimum biofilm inhibitory concentration (MBIC) value for all tested strains. The EEs exhibited various levels of inhibitory activities on the biofilm formation at the range of 31.5 to 250 μg/mL.

The effects of sub-inhibitory concentrations of the five efficacious EEs (licorice root, sage leaves, purple cornflower flower, purple cornflower stem, and slippery elm inner bark) on biofilm formation over 72 h incubation of the three *S. pyogenes* strains were quantified by 3-(4,5-dimethylthiazol-2-yl)-2,5-diphenyltetrazolium bromide (MTT) staining (Table 3). Penicillin G possessed the most pronounced inhibition of biofilm, with the lowest minimum biofilm inhibitory concentration (MBIC) values for all the strains. Plant extracts exhibited inhibitory activity on biofilm formation, ranging from 31.5–250 μg/mL. 

### 2.6. SEM Visualization of Fixed Biofilms of S. pyogenes

**Figure 4:**
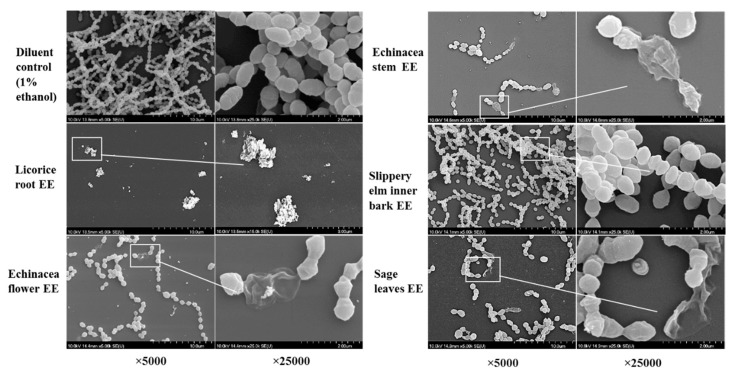
Scanning electron micrographs of *Streptococcus pyogenes* biofilms formed on the 96-well surface of sub-inhibitory concentrations of the ethanol extracts of licorice roots, purple coneflower flowers, purple coneflower stems, slippery elm inner barks, and sage leaves.

Changes in morphology and density of biofilm formed by *S. pyogenes* ATCC 19615 were observed after 72 h of treatment with above five EEs at their sub-biofilm inhibitory concentrations. The remaining biofilms/cells were visualized under SEM, and the results are shown in Figure 4. Surface morphological changes and bacterial cell densities were observed and compared with 1% ethanol diluent controls. The EE of licorice roots showed substantial biofilm inhibition by leaving only a cluster of dead cell debris. Other than the reduction of cell density, several morphological changes, such as changes of cocci shape, incomplete separation of cocci from the strep, an abnormal cell division, ruptured cell structure, and swelling were observed in the extract treatments. Furthermore, cell densities of treated samples have significantly reduced compared to untreated 

## 3. Discussion

Medicinal plant parts and their extracts have been used for treating various diseases for thousands of years. It has been shown that the presence of various phytochemicals of these plants provides beneficial effects including antimicrobial activities. This study assessed the ability of 14 EEs of selected common medicinal plants known to traditional healers in Canada for managing streptococcal pharyngitis. We have examined multiple anti-*S. pyogenes* attributes including planktonic growth inhibition, the time taken to give a bactericidal effect, morphological damage in cell wall/membrane, and biofilm inhibition. 

Application of medicinal plant parts as a source for therapeutic agents against *S. pyogenes* infections have been reported [8,9,10,11,43,44]. The existence of anti-microbial activity in particular parts of a plant species may be due to the presence of specific polyphenols, isoprenoids, alkaloids, steroids, and saponins [7,45]. Previous studies revealed a relationship between antimicrobial activity and the phytochemicals in the various medicinal plant extracts [10,45]. We have previously evaluated the total phenolic contents and total carotenoid contents of these EEs [46]. Moreover, in the present study, the extracts were further assessed for potential phytochemical constituents using UPLC-ESI-MS/MS (Table 1), and some of these constituents may associate with the antibacterial activity observed against *S. pyogenes* strains.

### 3.1. Bactericidal Effect and Cell Integrity Changes by Plant Extracts 

Extracts from licorice roots, sage leaves, purple coneflower flowers, purple coneflower stems, and slippery elm inner barks have remarkable inhibition activities against the planktonic growth of *S. pyogenes*. Hence, time to kill results are useful to determine the most efficacious extracts among those five extracts. Both licorice root and sage leaf extracts were the most efficacious bactericidal agents, which kill 99.99% of the initial bacterial load within 3 h exposure to the 2 × MIC. Usually, either whole aerial parts or sometimes roots of the purple coneflower species, such as *Echinacea angustifolia*, *E. pallida*, and *E. purpurea* are used to treat respiratory infections [9,47]. We have evaluated three different parts of purple coneflower (stem, flowers, and leaves) of *E. purpurea* for the first time against *S. pyogenes* to identify which aerial part has the highest activity against *S. pyogenes*. Among the tested parts of purple coneflower, only stem and flower extracts were found to be effective where leaf extract activity was not significant. Purple coneflower flower EE exhibited bactericidal effect against ATCC 19615 within 5 h of exposure to 2 × MIC, whereas it acquired 9 h to possess the similar bactericidal effect of stem EE. 

The present study investigated morphological changes, such as structural alterations of *S. pyogenes* after exposure to the effective EEs (licorice, sage, purple coneflower flower, and stem). It could be assumed that lower molecular mass compounds (such as carvacrol, thymol, caffeic acid, γ-terpinene, β-pinene, and α-pinene) enter through the peptidoglycan layer and act on the cytoplasmic membrane of *S. pyogenes*. Membrane structural changes, such as fluidity alteration, could also lead to a slight modification in the external surface of the cell wall and may be attributed to leakage of cytosolic fluids outside the cells. 

Antimicrobial potential of licorice root, sage leaves, and purple coneflower flowers and stems may be attributable to structure and their concentration of detected phytochemicals. The bioactive compounds, such as 1, 8-cineole, p-cymene, camphor, α-thujone, β-pinene, trans-caryophyllene, and β-thujone present in sage EE may have contributed to the antibacterial activity [48], and the present study has suggested its potential phytochemical profile (Table 1). These compounds are reported to possess several modes of action, such as disturbing the cytoplasmic membrane integrity of bacteria, affecting the electron transport chain, changing the pH homeostasis, disrupting the proton motive force, and coagulation of cell contents [48,49,50]. 

Licorice root showed an effect on biofilm, which could be due to the inhibition of cell integrity, as shown in Figure 2, leading to wall and membrane destruction and leakage. Licorice extract exhibited antimicrobial activity against both Gram-positive and Gram-negative bacteria. Most of the antimicrobial effects of licorice roots, as well as leaves, are reported due to isoflavonoid components (Table 1), particularly glabridin, glycyrrhizin, glabriol, hispaglabridin, B 4′-*O*-methylglabridin, and 3-hydroxyglabrol [51]. Furthermore, the effect of licorice roots against some oral pathogens can be attributed to several mechanisms; for example, glycyrrhizin and glycyrrhizic acid have displayed inhibition of bacterial growth [52]. However, a previous study reported the side effects of licorice supplementation, mainly elevated blood pressure [53]; therefore, further investigations are required before making any recommendations.

Different varieties of thyme and oregano extracts are known to possess antimicrobial potential [31,41,48]. Both plant leaves and young shoots are used in various food preparations as flavor enhancers as well as in herbal remedies. Although the mechanisms of action of oregano and thyme extracts and oils were not thoroughly studied, previous studies have suggested that antimicrobial activity may be due to the presence of phytochemicals, including terpenes, thymol, eugenol, flavones, glycosides of phenolic monoterpenoids and aliphatic alcohols [48,54]. The phytochemicals identified in oregano and thyme EEs in the current study confirms the previous observations.

Destruction of the *S. pyogenes* bacterial cell wall and cell membrane as observed in TEM images could be one of the probable modes of action for the identified phytochemicals present in the efficacious extracts. The leakage of the cytoplasmic content and genetic materials of the treated *S. pyogenes* underwent morphological changes leading ultimately to cell death. Several studies have shown the ability of phenolic acids for their effects on the cell wall as well as cell membrane destructions on Gram-positive bacteria. For example, effects of phenolic acids such as *p*-coumaric acid, caffeic acid, ferulic acid, *p*-hydroxybenzoic acid, protocatechuic acid, gallic acid, and syringic acids on cell membrane permeability of lactic acid bacteria were revealed [55]. Moreover, TEM images and other experimental results have shown the ability of *Boesenbergia rotunda* (L.) Mansf. extract (Chinese keys/Chinese ginger) on damaging the cell membrane and cell wall peptidoglycan layer, leading to leakage of intracellular material in β-lactam-resistant staphylococci [56].

### 3.2. Anti-Biofilm Formation Ability of Plant Extracts 

Development of biofilm is a key mechanism involved in *S. pyogenes* virulence during pharyngitis infections, which assures superior survival and protection from host defensive mechanisms, antibiotics, and other environmental fluctuations [57,58]. It has been proposed that the treatment challenges of antibiotics in the eradication of *S. pyogenes* may be due to the presence of bacterial biofilm, also known as communities, attached to host cell surfaces and covered by extra polymeric substances (EPS) [59,60,61,62]. These EPS matrix surrounded by live bacterial cells are consist of extracellular polysaccharides, DNA, and proteins [61]. Several previous studies have been reported that medicinal plants prevent the different steps of biofilm in various Gram-positive bacteria, including *S. pyogenes* [63,64]. 

MBIC values represented the metabolic activity of biofilm biomass, and results of the current study indicated that the plant extracts possibly interfered during *S. pyogenes* biofilm formation at some unknown stage rather than destroying them after formation. It is essential to explore the effect on biofilm is due to inhibition of biofilm-forming pathways or killing of planktonic cells. However, time to kill curves performed in the presence of sub-inhibitory concentrations of five EEs showed a considerably altered pattern of growth curve (Figure 3), indicating a delay or stoppage in the exponential phase. Observations of SEM results (Figure 4) of the cell density and morphology of biofilms further confirmed the destructive activities of extracts on biofilms. When treated with extracts *S. pyogenes* did not form thick biofilm layers. Penicillin G is highly effective even at sub-MBIC, therefore, this may be explained by the reduction in *S. pyogenes* biofilm due to the killing of its planktonic cells. 

Fuqua, et al. [65] suggested that phytochemicals of medicinal plant suppress the expression of genes responsible for bacterial pathogenicity by interfering with the formation of biofilm. However, plant extracts comprise a large number of components, and it is expected that their mechanisms of action involve several targets in the *S. pyogenes* cells rather than a single mechanism [50]. We have previously performed cell viability assay to assess the cytotoxicity of these extracts on human tonsil epithelial cell line and demonstrated that non-toxic concentrations to human normal cell line could be used to develop natural health products or antibacterial therapy in future [46].

## 4. Materials and Methods 

### 4.1. Collection of Plant Materials

Twelve different herbal plants from Canadian traditional medicine were selected for the study (Table 4). Purple coneflower (Echinacea), geranium, sage, oregano, and thyme were collected from the university’s herbal garden, Faculty of Agriculture, Dalhousie University, during the flowering period. A taxonomist, Jeff Morton, authenticated plants and plant specimens were deposited in the A.E. Roland herbarium, Department of Plant, Food, and Environmental Sciences, Faculty of Agriculture, Dalhousie University, Canada. Fresh ginger (Chinese and Canadian) and dried clove flower buds were purchased from Halifax (NS, Canada) supermarkets. Danshen roots were received from Green Man Botanicals (Gaspereau Mountain, NS, Canada). Dry powders of barberry root, licorice root, slippery elm, and red elm inner bark, and olive leaves were purchased (Mother Earth Natural Health, Ottawa, ON, Canada). Samples were oven dried at 40 °C, and ground powder samples were stored in airtight bottles at −80 °C until being used for further extraction steps.

### 4.2. Preparation of Ultrasonic-Assisted Ethanol Extracts (EE)

The EEs were prepared similarly to a previously described method [9]. Briefly, 1:10 ratio of grounded dried plant powder and 95% ethanol mixtures were kept in a sonication bath (750 D, VWR, West Chester, PA, USA) at 35 °C for 45 min (15 min × 3, 10 min interval; 40 kHz frequency and 150W ultrasonic power). The filtrates were collected by filtering through a vacuum pump and were evaporated to dryness using a rotary evaporator (R-200, Buchi, Flawil, Switzerland) at 45 °C for 20 to 30 min. Dried remained solids were collected by dissolving them in anhydrous ethanol and dried completely under the nitrogen evaporator (N-EVAP^TM^, Organomation Association Inc., Berlin, NJ, USA). They were stored in airtight amber glass bottles at −80 °C until use.

### 4.3. Phytochemical Analysis: Mass Spectrometric Characterization

Total phenolic content and total carotenoid content of these extracts were previously reported [46]. Full scan mode of ultra-performance liquid chromatographic-electrospray ionization-tandem mass spectrophotometry (UPLC-ESI-MS/MS) was performed for characterization of potential phytochemicals in the extracts. UPLC was directly interfaced with a High Definition MS System (Waters Xevo TQ-Smicro, Waters Corporation, Milford, CT, USA) equipped with an electrospray ion (ESI) source operating in negative ion mode. The optimal ionization conditions were adjusted as follows: ESI negative mode, a capillary voltage of 2.0 kV, sampling cone voltage of 25.0 V, and extraction cone voltage was 3.5 V. The source temperature was set at 150 °C, desolvation gas temperature was 450 °C, while the cone gas flow and desolvation gas flow were 100 L/h and 1000 L/h, respectively. Full scan mass acquisitions were performed in negative ion mode by scanning the *m*/*z* range from 100 to 1100 Da. Data were collected in centroid mode, and mass was corrected during acquisition using an external reference (Lock-Spray™) comprising a 2.1 × 100 mm C_18_ columns (UPLC^®^BEH C18, Waters Corporation, USA). All solvents were of HPLC-grade. The samples were filtered through a nylon 0.22-μm membrane (Nalgene™, Rochester, NY, USA), and were auto injected into the column. The mobile phase was a mixture of two solvent compositions, solvent A (0.1% formic acid in water) and solvent B (0.1% formic acid in acetonitrile). The program was started with 94.0% solvent A flow of 0.3 mL/min and total run time was set to 12 min. The separation gradient used was: 2 min (83.5% A), 2.6 min (83.0% A), 3.1 min (82.5% A), 4 min (81.5% A), 4.7 min (80.0% A), 6.6 min (20.0% A), 8.2 min (20.0% A), and 12 min (94.0%). 

Identification of the major constituents was carried out based on the full scan performed in the range of 100 to 1100 Da using UPLC-ESI-MS/MS. The deprotonated (M − H^+^)^−^ ions identified using the full scan mode was used to create 51 selected ion monitoring (SIM) channels (an example of sage leaves extract is presented in Supplementary Figure S1). The samples were re-run in SIM mode to confirm the abundance of each identified constituent and the matching of the retention times of full scan and SIM modes. The identified compounds in each plant extract were also verified with the available literature of their deprotonated ions (Table 2).

### 4.4. Bacterial Strains and Culture Maintenance

Three strains of *S. pyogenes* were used in this study. Two *S. pyogenes* reference strains were obtained from the American Type Culture Collection (ATCC), ATCC 19615, and ATCC 49399. Inoculums were prepared according to the manufacturer’s instructions. A clinical isolate, obtained from a pharyngitis patient, was kindly provided by Dr. Ross Davidson and was cultured similarly. All strains were stored at −80 °C in brain heart infusion (BHI) (Oxoid Ltd. Nepean, ON, Canada) supplemented with 20% glycerol. Strains were cultured on BHI agar plates and were maintained for seven days at 35 ± 2 °C. One or two colonies from these cultures were inoculated onto BHI broth and incubated at 35 ± 2 °C for 24 h before the experiment and were diluted to a 1 × 10^6^ colony forming units (CFU)/mL with saline water (0.85% NaCl, pH = 7.0 ± 0.1). 

### 4.5. Anti-Bacterial Activity

#### 4.5.1. Determination of Minimum Inhibitory Concentrations (MICs)

Inhibition of bacterial growth was determined following the clinical and laboratory standards institute (CLSI) [66] standard broth microdilution method. In a 96-well plate, 100 µL of the bacterial suspension in BHI broth was added to wells containing 100 µL of plant extracts in broth. Serial two-fold dilutions of each of the plant extracts or drug (concentrations ranging from 0.75 to 1000 μg/mL for EEs and 0.0004 to 0.5 μg/mL for penicillin G) were made. Each plate included positive controls (bacteria without antimicrobials), negative controls (media), and diluent controls (100% ethanol). After incubation for 24 h at 35 ± 2 °C, the growth of planktonic bacteria was determined by measuring absorbance at OD 600 nm, using a microplate reader (Epoch^TM^, Biotek, Winooski, VT, USA). The MIC values, the lowest concentration of test compounds inhibiting visible bacterial growth (visual method), and a significant change of absorbance compared to the positive control (*p* ≤ 0.05) in the spectrophotometric method were recorded. When there was more than 90% inhibition, the corresponding concentration was considered as the MIC (specifically MIC 90).

#### 4.5.2. Determination of Minimum Bactericidal Concentrations (MBCs)

The 33 μL of suspension from each well showing no visible growth was spread out on a BHI agar plate. Colony growth was observed after 24 h incubation at 35 ± 2 °C.

### 4.6. Time to Kill Assay

The time taken to give a bactericidal activity after exposure of extracts were assessed using time-kill curves [11]. The ATCC 19615 bacterial suspension (1 × 10^6^ CFU/mL) was added to BHI broth containing EEs at 1/2 × MIC, MIC, and 2 × MIC, or dimethyl sulfoxide (DMSO) (Sigma-Aldrich Ltd., Oakville, ON, Canada) vehicle alone (as a negative control) in 96-well plates. The plates were incubated at 35 ± 2 °C, and bacterial growth was monitored over different incubation periods at different time intervals, based on the preliminary assay results. Licorice root EE, purple coneflower EE, and sage flowering shoots EE were tested over 6-h period at 2 h intervals. Purple coneflower stem EE and slippery elm inner bark EEs were taken over 24-h period at 3 h intervals for agar plating. 

### 4.7. TEM Observations of Bacterial Cell Wall Damage 

Bacteria (1 × 10^6^ CFU/mL) were incubated along with the same volume of BHI containing compound or control (at sub-MIC concentration) at 37 °C for 16 h. Then, 5–10 mL from each treatment was centrifuged at 10,000 rpm for 10 min, and the media was discarded. Cells were washed three times with phosphate buffered saline (PBS) (pH 7.4) (Sigma-Aldrich Ltd., Oakville, ON, Canada) for 15 min each. After discarding PBS (pH 7.4), the sample pellets were fixed with 0.1 M sodium cacodylate trihydrate solution with 2% glutaraldehyde for 2 h (minimum). Following 2 h immersion, the sample wells were rinsed (3 times × 10 min minutes each) with 0.1 M sodium cacodylate buffer. Then, the second fixation was carried out by submerging samples with 1% osmium tetroxide solution in 0.1 M cacodylate for 2 h at room temperature. Then, samples were rinsed well with distilled water and were placed in 0.25% uranyl acetate at 4 °C overnight. The fixed cell pellets were dehydrated with graduated ascending acetone gradients series (50%, 70%, 95%, and 100%) and were dried in 100% acetone (10 min with each concentration repeated twice except the first (50:50) and last one (100:0), which is washed only one time). The samples were infiltrated with Epon Araldite Resin as a 3:1 ratio of dried 100% acetone: resin for 3 h; 1:3 ratio of dried 100% acetone: resin overnight; and finally, 100% Epon Araldite Resin for 2 × 3 h. Samples embedded in 100% Epon Araldite Resin were placed in a 60 °C oven for 48 h to cure (harden) properly. Thin sections were cut using a microtome (Reichert-Jung Ultracut E Ultramicrotome, EquipNet, Inc., Peabody, MA, USA) with a diamond knife (approximately 100 nm thin). Thin cuts were placed on 300 mesh copper grids, which were then stained as follows: 2% aqueous uranyl acetate for 10 min, distilled water rinse for 2 × 5 min, lead citrate for 4 min, a quick rinse with distilled water, and then air dried. Samples were viewed using a transmission electron microscope (JEM 1230, JEOL USA, Inc., Peabody, MA, USA) at 80 kV and images were captured using a digital camera (ORCA-HR, Hamamatsu Photonics, Tokyo, Japan).

### 4.8. Anti-Biofilm Formation Activity

#### 4.8.1. Determination of Minimum Biofilm Inhibitory Concentration (MBIC)

The anti-biofilm formation activity was evaluated using 3-(4,5-dimethylthiazol-2-yl)-2,5-diphenyltetrazolium bromide (MTT) (Life Technologies, Burlington, ON, Canada) assay, as described previously [11] for five EEs, solvent controls, and penicillin G. 

#### 4.8.2. SEM Visualization for Biofilm Morphology

Sub-inhibitory biofilm inhibitory concentration of selected extracts was fixed and visualized in a field emission gun scanning electron microscope (Hitachi FEG-SEM 4700, Hitachi Ltd., Tokyo, Japan), as described in Wijesundara and Rupasinghe [11]. 

### 4.9. Statistical Analysis

The complete randomized design was used as the experimental design. All the experiments were performed in triplicate and independently, three times. One-way ANOVA analysis was carried out by using Minitab 17.0 statistical software, and statistical differences (*p* ≤ 0.05) between means of pairs were resolved using confidence intervals using Tukey’s tests. Results were expressed as a mean ± standard deviation.

## 5. Conclusions

We report the inhibitory effect on the growth and biofilm formation of *S. pyogenes* by phytochemical-rich ethanol extracts derived from selected Canadian traditional medicinal plants, emphasizing their importance as an alternative treatment. We have found that the extracts of sage leaf, purple coneflower flower, and licorice root EEs can significantly reduce the planktonic growth and biofilm formation of *S. pyogenes*. Potential mechanism of these efficacious four EEs could be due to their impact on cell wall and membrane degradation, which decrease the planktonic growth reducing bacteria population to form the biofilm. However, further investigations are required to determine changes in cell content, their leakage, and specific gene expression to confirm the mechanism of the cell wall and membrane disruption as well as biofilm formation. As well, the standardization of commercial herbal extract preparation and chemical characterization of the putative phytochemicals are essential for the advancement of natural health products for managing streptococcal pharyngitis. 

## Figures and Tables

**Table 1:** Phytochemical profile of the herbal plant parts used in this study.

Extract Name	Potential Major Phytochemicals ([M − H^+^]^−^, RT)	References
Barberry roots	Jatrorrhizine (337.38, 8.04), Palmatine (341.40, 9.28), Berbamine (608.7, 7.79), Quercetin (301.23, 7.49), Rutin (609.52, 4.73), and Kaempferol (285.23, 6.50).	[12,13,14]
Clove flower buds	Eugenol (163.20, 4.40), β-Ocimene (135.25, 4.25), p-Allyl phenol (133.17, 0.84), and Kaempferol (285.23, 6.50).	[15,16]
Danshen roots *	NI in this study Danshenxinkun A, Protocatechuic acid, Caffeic acid, Rosmarinic acid, Tanshinone VI, 17-Hydroxycryptotanshinone, Salvianolic acid, and Salvianolic acid A.	[17,18]
Purple coneflower flowers	Caftaric acid (311.23, 2.44), Chlorogenic acid (353.31, 2.78), Caffeic acid (179.16, 3.27), Cynarin (515.46, 6.02), Echinacoside (785.73, 6.56), and Cichoric acid (473.37, 4.78).	[19,20,21,22]
Purple coneflower leaves	Chlorogenic acid (353.31, 2.78), Caffeic acid (179.16, 3.27), Cynarin (515.46, 6.02) Echinacoside (785.73, 6.56), and Cichoric acid (473.37, 4.78).	[19,22]
Purple coneflower stems	Caftaric acid (311.23, 2.44), Chlorogenic acid (353.31, 2.78) and Cichoric acid (473.37, 4.78), Quercetin (301.23. 7.49), Caryophyllene oxide (375.45, 7.40), and Liquirtin (419.39, 6.21).	[19,22]
Geranium leaves	Geraniol (151.24, 6.62).	[23]
Ginger rhizome	Gingerol (273.38, 7.18), Galanolactone (317.45, 8.94), Zingerone (193.22, 4.96), α-Humulene (203. 24, 2.50), and α or β-Caryophyllene (203.36, 2.52).	[24]
Licorice root	Naringin (579.54, 6.28), Asparegene (131.12, 1.63), Rosmarinic acid (359.31, 6.23) and Liquirtin (419.39, 6.21).	[25,26,27]
Oregano flowering shoots	p-Cymene and/or Borneol and/or 1,8 Cineole, (153.24, 2.20), Thymohydroquinone (165.22, 7.17), Rosmarinic acid (359.31, 6.23) and Naringin (579.54, 6.28).	[11,28,29,30,31]
Olive leaves *	NI in this study. Gallic acid, Hydroxytyrosol, Tyrosol, Caffeic acid, p-Coumaric, Oleuropein, Luteolin, and Quercetin.	[32]
Sage leaves	Borneol and/or Linalool and/or α-Terpineol and/or β-Pinene (135.24, 3.05 or 4.01), Asparagine (131.12, 3.65), γ-Terpinene and/or Mycrene and/or β-Pinene and/or α-Pinene (135.24, 4.24 and Kaempferol (285.23, 6.50).	[11,33,34,35,36,37]
Slippery elm inner barks	Oleanolic acid (455.71, 7.2), Ursolic acid and/or Betulinic acid (455.71, 7.50 or 9.6) and β-carotene (535.87, 5.29).	[38]
Thyme flowering shoots	Thymol and/or Carvacrol (149.21, 6.65), Thymohydroquinone (165.22, 7.17), γ-Terpinene, Mycrene, and α-Pinene (135.24, 4.24) and Kaempferol (285.23, 6.50).	[39,40,41,42]

Constituent phytochemical profiles of ethanol extracts were assessed using ultra-performance liquid chromatographic-electrospray ionization-tandem mass spectrophotometry. [M − H^+^]^−^: deprotonated molecular mass, RT: retention time and NI: not identified. * Danshen roots and olive ethanol extracts were not assessed due to the less availability of dry extracts for chromatography sample preparation. However, their potential phytochemicals reported in the literature was included in the Table 1.

**Table 2:** Minimum inhibitory concentrations (MICs) and minimum bactericidal concentrations (MBCs) of selected ethanol extracts against *Streptococcus pyogenes* strains.

Plant Source/Reference	ATCC 19615			ATCC 49399			Clinical Isolate		
	MIC (μg/mL)	MBC (μg/mL)	MBC/MIC	MIC (μg/mL)	MBC (μg/mL)	MBC/MIC	MIC (μg/mL)	MBC (μg/mL)	MBC/MIC
Barberry Roots	250	500	2	250	500	2	250	500	2
Clove Flower Buds	500	1000	2	500	1000	2	500	1000	2
Dan shen Roots	250	>250	-	250	>250	-	NA	NA	-
Purple Coneflower Flower	62.5	125	2	62.5	125	2	62.5	125	2
Purple Coneflower Leaves	>1000	>1000	-	>1000	>1000	-	NA	NA	-
Purple Coneflower Stem	62.5	125	2	62.5	125	2	125	250	2
Geranium Leaves	>1000	>1000	-	>1000	>1000	-	NA	NA	-
Ginger Rhizomes	>1000	>1000	-	>1000	>1000	-	NA	NA	-
Licorice Roots	62.5	125	2	62.5	125	2	62.5	125	2
Olive Leaves	>1000	>1000	-	>1000	>1000	-	NA	NA	-
Oregano Flowering Shoots	500	1000	2	500	1000	2	500	1000	2
Sage Leaves	62.5	125	2	62.5	125	2	62.5	125	2
Slippery elm Inner barks	62.5	125	1	62.5	125	2	62.5	125	1
Thyme Flowering shoots	500	1000	2	500	1000		500	1000	-
Penicillin	0.008	0.016	2	0.008	0.016	2	0.008	0.016	2

**Table 3:** Minimum biofilm inhibitory concentration (MBIC) of selected medicinal extracts against *Streptococcus pyogenes*.

Plant	MBIC (μg/mL)		
	ATCC 19615	ATCC 49399	Clinical
**Licorice Roots**	250 (4 × MIC)	250 (4 × MIC)	62.5 (1 × MIC)
**Purple Coneflower Stems**	125 (2 × MIC)	250 (4 × MIC)	250 (2 × MIC)
**Purple Coneflower Flowers**	125 (2 × MIC)	31.5 (1 × MIC)	62.5 (1 × MIC)
**Sage Leaves**	125 (2 × MIC)	125 (2 × MIC)	125 (2 × MIC)
**Slippery elm Inner Bark**	62.5 (1 × MIC)	62.5 (1 × MIC)	125 (2 × MIC)
**Penicillin G**	0.0156 (2 × MIC)	0.0625 (8 × MIC)	0.0625 (8 × MIC)

*MIC: Minimum inhibitory concentration*.

**Table 4:** The details of the herbal plants used in the study.

Plant Name		Family	Parts Used	Voucher No.	GPS Location of Harvested Area
Common	Botanical				
**Barberry**	*Berberis vulgaris* L.	Berberidaceae	Root	-	Purchased
**Clove**	*Syzygium aromaticum* L.	Myrtaceae	Flower bud	-	Purchased
**Danshen**	*Salvia miltiorrhiza* Bunge.	Lamiaceae	Roots	-	Purchased
**Echinacea/Purple cone-flower**	*Echinacea purpurea* L.	Asteraceae	Leaves, stem, flower	13009	45°22′20.8″ N 63°15′43.8″ W
**Ginger**	*Zingiber officinale* L.	Zingiberaceae	Rhizome	-	Purchased
**Licorice**	*Glycyrrhiza glabra* L.	Papilionaceae	Root	-	Purchased
**Oregano**	*Origanum vulgare* L.	Lamiaceae	Flowering shoots and leaves	13012	45°22′23.3″ N 63°15′45.2″ W
**Olive**	*Olea europeus* L.	Oleaceae	Leaves	-	Purchased
**Rose geranium**	*Pelargonium graveolens* L.	Geraniaceae	Leaves	13010	45°22′23.3″ N 63°15′45.2″ W
**Sage**	*Salvia officinalis* L.	Lamiaceae	leaves, root	13011	45°22′23.3″ N 63°15′45.2″ W
**Slippery elm**	*Ulmus rubra* Muhl.	Ulmaceae	Inner bark	-	Purchased
**Thyme**	*Thymus vulgaris* L.	Lamiaceae	Flowering shoots and leaves	13013	45°22′23.3″ N 63°15′45.2″ W

## Supplementary Materials

The following are available online at https://www.mdpi.com/1420-3049/24/6/1165/s1, Figure S1: The full scan spectra and SIM chromatograms of sage leaves ethanol extracts of UPLC-ESI-MS/MS analysis.

## References

[1] Carapetis J.R., Steer A.C., Mulholland E.K., Weber M. (2005). The global burden of group A streptococcal diseases. Lancet Infect Dis..

[2] Bisno A.L., Peter G.S., Kaplan E.L. (2002). Diagnosis of strep throat in adults: Are clinical criteria really good enough?. Clin. Infect Dis..

[3] Martin J.M. (2015). The Mysteries of Streptococcal Pharyngitis. Curr. Treat Options Pediatr..

[4] Shulman S.T., Bisno A.L., Clegg H.W., Gerber M.A., Kaplan E.L., Lee G., Martin J.M., Van Beneden C. (2012). Clinical practice guideline for the diagnosis and management of group A streptococcal pharyngitis: 2012 update by the Infectious Diseases Society of America. Clin. Infect Dis..

[5] Llor C., Bjerrum L. (2014). Antimicrobial resistance: Risk associated with antibiotic overuse and initiatives to reduce the problem. Ther. Adv. Drug Saf..

[6] Palla A.H., Khan R.A., Gilani A.H., Marra F. (2012). Over prescription of antibiotics for adult pharyngitis is prevalent in developing countries but can be reduced using McIsaac modification of Centor scores: A cross-sectional study. BMC Pulm. Med..

[7] Cowan M.M. (1999). Plant products as antimicrobial agents. Clin. Microbiol. Rev..

[8] Abachi S., Lee S., Rupasinghe H.P.V. (2016). Molecular mechanisms of inhibition of *streptococcus* species by phytochemicals. Molecules.

[9] Sharma S.M., Anderson M., Schoop S.R., Hudson J.B. (2010). Bactericidal and anti-inflammatory properties of a standardized Echinacea extract (Echinaforce): Dual actions against respiratory bacteria. Phytomedicine.

[10] Zuo G.-Y., Yang C.-X., Han J., Li Y.-Q., Wang G.-C. (2018). Synergism of prenylflavonoids from *Morus alba* root bark against clinical MRSA isolates. Phytomedicine.

[11] Wijesundara N.M., Rupasinghe H.P.V. (2018). Essential oils from *Origanum vulgare* and *Salvia officinalis* exhibit antibacterial and anti-biofilm activities against *Streptococcus pyogenes*. Micro. Pathogenesis.

[12] Abd El-Wahab A.E., Ghareeb D.A., Sarhan E.E., Abu-Serie M.M., El Demellawy M.A. (2013). In vitro biological assessment of Berberis vulgaris and its active constituent, berberine: Antioxidants, anti-acetylcholinesterase, anti-diabetic and anticancer effects. BMC Complement. Altern. Med..

[13] Kukula-Koch W., Aligiannis N., Halabalaki M., Skaltsounis A.-L., Glowniak K., Kalpoutzakis E. (2013). Influence of extraction procedures on phenolic content and antioxidant activity of Cretan barberry herb. Food Chem..

[14] Suau R., Rico R., López-Romero J.M., Nájera F., Cuevas A. (1998). Isoquinoline alkaloids from *Berberis Vulgaris* subsp. Australis. Phytochemistry.

[15] Lee K.-G., Shibamoto T. (2001). Antioxidant property of aroma extract isolated from clove buds [*Syzygium aromaticum* (L.) Merr. et Perry]. Food Chem..

[16] de Oliveira M.S., da Costa W.A., Pereira D.S., Botelho J.R.S., de Alencar Menezes T.O., de Aguiar Andrade E.H., da Silva S.H.M., da Silva Sousa Filho A.P., de Carvalho R.N. (2016). Chemical composition and phytotoxic activity of clove (*Syzygium aromaticum*) essential oil obtained with supercritical CO2. J. Supercrit. Fluid..

[17] Cao J.-L., Wei J.-C., Hu Y.-J., He C.-W., Chen M.-W., Wan J.-B., Li P. (2016). Qualitative and quantitative characterization of phenolic and diterpenoid constituents in Danshen (*Salvia miltiorrhiza*) by comprehensive two-dimensional liquid chromatography coupled with hybrid linear ion trap Orbitrap mass. J. Chromatogr. A.

[18] Yang M., Liu A.H., Guan S.H., Sun J.H., Xu M., Guo D. (2006). Characterization of tanshinones in the roots of *Salvia miltiorrhiza* (Dan-shen) by high-performance liquid chromatography with electrospray ionization tandem mass spectrometry. Rapid Commun. Mass Sp..

[19] Hudec J., Burdová M., Kobida L.U., Komora L., Macho V., Kogan G., Turianica I., Kochanová R., Ložek O., Habán M. (2007). Antioxidant Capacity Changes and Phenolic Profile of *Echinacea purpurea*, Nettle (*Urtica dioica* L.), and Dandelion (*Taraxacum officinale*) after Application of Polyamine and Phenolic Biosynthesis Regulators. J. Agric. Food Chem..

[20] Kim H.-O., Durance T.D., Scaman C.H., Kitts D.D. (2000). Retention of Caffeic Acid Derivatives in Dried *Echinacea purpurea*. J. Agric. Food Chem..

[21] Tsai Y.-L., Chiou S.-Y., Chan K.-C., Sung J.-M., Lin S.-D. (2012). Caffeic acid derivatives, total phenols, antioxidant and antimutagenic activities of *Echinacea purpurea* flower extracts. LWT-Food Sci. Technol..

[22] Vimalanathan S., Kang L., Amiguet V.T., Livesey J., Arnason J.T., Hudson J. (2005). *Echinacea purpurea* aerial parts contain multiple antiviral compounds. Pharm. Biol..

[23] Hsouna A.B., Hamdi N. (2012). Phytochemical composition and antimicrobial activities of the essential oils and organic extracts from *pelargonium graveolens* growing in Tunisia. Lipids Health Dis..

[24] Račková L., Cupáková M., Tažký A., Mičová J., Kolek E., Košt’álová D. (2013). Redox properties of ginger extracts: Perspectives of use of *Zingiber officinale* Rosc. as antidiabetic agent. Interdiscip. Toxicol..

[25] Çakmak Y.S., Aktumsek A., Duran A. (2012). Studies on antioxidant activity, volatile compound and fatty acid composition of different parts of *Glycyrrhiza echinata* L.. EXCLI J..

[26] Montoro P., Maldini M., Russo M., Postorino S., Piacente S., Pizza C. (2011). Metabolic profiling of roots of liquorice (*Glycyrrhiza glabra*) from different geographical areas by ESI/MS/MS and determination of major metabolites by LC-ESI/MS and LC-ESI/MS/MS. J. Pharm. Biomed..

[27] Simons R., Vincken J.P., Bakx E.J., Verbruggen M.A., Gruppen H. (2009). A rapid screening method for prenylated flavonoids with ultra-high-performance liquid chromatography/electrospray ionization mass spectrometry in licorice root extracts. Rapid Commun. Mass Sp..

[28] Koldas S., Demirtas I., Ozen T., Demirci M.A., Behcet L. (2015). Phytochemical screening, anticancer and antioxidant activities of *Origanum vulgare* L. ssp viride (Boiss.) Hayek, a plant of traditional usage. J. Sci. Food Agric..

[29] Ličina B.Z., Stefanović O.D., Vasić S.M., Radojević I.D., Dekić M.S., Čomić L.R. (2013). Biological activities of the extracts from wild growing *Origanum vulgare* L.. Food Control.

[30] Milos M., Mastelic J., Jerkovic I. (2000). Chemical composition and antioxidant effect of glycosidically bound volatile compounds from oregano (*Origanum vulgare* L. ssp. hirtum). Food Chem..

[31] Teixeira B., Marques A., Ramos C., Serrano C., Matos O., Neng N.R., Nogueira J.M., Saraiva J.A., Nunes M.L. (2013). Chemical composition and bioactivity of different oregano (*Origanum vulgare*) extracts and essential oil. J. Sci. Food Agric..

[32] Maalej A., Bouallagui Z., Hadrich F., Isoda H., Sayadi S. (2017). Assessment of *Olea europaea* L. fruit extracts: Phytochemical characterization and anticancer pathway investigation. Biomed. Pharmacother..

[33] Exarchou V., Nenadis N., Tsimidou M., Gerothanassis I.P., Troganis A., Boskou D. (2002). Antioxidant Activities and Phenolic Composition of Extracts from Greek Oregano, Greek Sage, and Summer Savory. J. Agric. Food Chem..

[34] Hamidpour M., Hamidpour R., Hamidpour S., Shahlari M. (2014). Chemistry, Pharmacology, and Medicinal Property of Sage (*Salvia*) to Prevent and Cure Illnesses such as Obesity, Diabetes, Depression, Dementia, Lupus, Autism, Heart Disease, and Cancer. J. Tradit. Complement. Med..

[35] Šulniūtė V., Pukalskas A., Venskutonis P.R. (2017). Phytochemical composition of fractions isolated from ten *Salvia* species by supercritical carbon dioxide and pressurized liquid extraction methods. Food Chem..

[36] Veličković D.T., Milenović D.M., Ristić M.S., Veljković V.B. (2006). Kinetics of ultrasonic extraction of extractive substances from garden (*Salvia officinalis* L.) and glutinous (*Salvia glutinosa* L.) sage. Ultrason. Sonochem..

[37] Yilar M., Kadioglu I., Telci I. (2018). Chemical Composition and Antifungal Activity of *Salvia Officinalis* (L.), S. Cryptantha (Montbret Et Aucher Ex Benth.), S. Tomentosa (Mill.) Plant Essential Oils and Extracts. Fresen. Environ. Bull..

[38] Barsett H., Smestad Paulsen B. (1992). Separation, isolation and characterization of acidic polysaccharides from the inner bark of *Ulmus glabra* Huds. Carbohyd. Polym..

[39] Al Hashmi L.S., Hossain M.A., Weli A.M., Al-Riyami Q., AlSabahi J.N. (2013). Gas chromatography–mass spectrometry analysis of different organic crude extracts from the local medicinal plant of *Thymus vulgaris* L.. Asian Pac. J. Trop. Med..

[40] Habashy N.H., Abu Serie M.M., Attia W.E., Abdelgaleil S.A.M. (2018). Chemical characterization, antioxidant and anti-inflammatory properties of Greek *Thymus vulgaris* extracts and their possible synergism with Egyptian *Chlorella vulgaris*. J. Funct. Foods.

[41] Köksal E., Bursal E., Gülçin İ., Korkmaz M., Çağlayan C., Gören A.C., Alwasel S.H. (2017). Antioxidant activity and polyphenol content of Turkish thyme (*Thymus vulgaris*) monitored by liquid chromatography and tandem mass spectrometry. Int. J. Food Prop..

[42] Pereira O.R., Peres A.M., Silva A.M.S., Domingues M.R.M., Cardoso S.M. (2013). Simultaneous characterization and quantification of phenolic compounds in Thymus x citriodorus using a validated HPLC–UV and ESI–MS combined method. Food Res. Int..

[43] Zhang B., Wijesundara N.M., Abbey L., Rupasinghe H.P.V. (2017). Growing medium amendments effect on growth, secondary metabolites and anti-streptococcal activity of two species of Plectranthus. J. Appl. Res. Med. Aromat. Plants.

[44] Mohd A., Rosina K., Rupasinghe H.P.V. (2018). Application of Medicinal Plants as a Source for Therapeutic Agents Against *Streptococcus pyogenes* Infections. Curr. Drug Metab..

[45] Zacchino S.A., Butassi E., Liberto M.D., Raimondi M., Postigo A., Sortino M. (2017). Plant phenolics and terpenoids as adjuvants of antibacterial and antifungal drugs. Phytomedicine.

[46] Wijesundara N.M., Sekhon-Loodu S., Rupasinghe H.P.V. (2017). Phytochemical-rich medicinal plant extracts suppress bacterial antigens-induced inflammation in human tonsil epithelial cells. Peer J..

[47] Kumar K.M., Ramaiah S. (2011). Pharmacological importance of *Echinacea purpurea*. Int. J. Pharm. Biol. Sci..

[48] Fournomiti M., Kimbaris A., Mantzourani I., Plessas S., Theodoridou I., Papaemmanouil V., Kapsiotis I., Panopoulou M., Stavropoulou E., Bezirtzoglou E.E. (2015). Antimicrobial activity of essential oils of cultivated oregano (*Origanum vulgare*), sage (*Salvia officinalis*), and thyme (*Thymus vulgaris*) against clinical isolates of *Escherichia coli*, *Klebsiella oxytoca*, and *Klebsiella pneumoniae*. Microb. Ecol. Health Dis..

[49] Abu-Darwish M.S., Cabral C., Ferreira I.V., Goncalves M.J., Cavaleiro C., Cruz M.T., Al-bdour T.H., Salgueiro L. (2013). Essential oil of common sage (*Salvia officinalis* L.) from Jordan: Assessment of safety in mammalian cells and its antifungal and anti-inflammatory potential. Biomed. Res. Int..

[50] Burt S. (2004). Essential oils: Their antibacterial properties and potential applications in foods--a review. Int. J. Food Microbiol..

[51] Fu Y., Chen J., Li Y.J., Zheng Y.F., Li P. (2013). Antioxidant and anti-inflammatory activities of six flavonoids separated from licorice. Food Chem..

[52] Sedighinia F., Safipour Afshar A., Soleimanpour S., Zarif R., Asili J., Ghazvini K. (2012). Anti-bacterial activity of *Glycyrrhiza glabra* against oral pathogens: An in vitro study. Avicenna J. Phytomed..

[53] Zadeh J.B., Kor Z.M., Goftar M.K. (2014). Licorice (*Glycyrrhiza glabra* Linn) as a valuable medicinal plant. Int. J. Adv. Biol. Biomed. Res..

[54] Nzeako B.C., Al-Kharousi Z.S., Al-Mahrooqui Z. (2006). Antimicrobial activities of clove and thyme extracts. Sultan Qaboos Univ. Med. J..

[55] Campos F.M., Couto J.A., Figueiredo A.R., Toth I.V., Rangel A.O., Hogg T.A. (2009). Cell membrane damage induced by phenolic acids on wine lactic acid bacteria. Int. J. Food Microbiol..

[56] Teethaisong Y., Pimchan T., Srisawat R., Hobbs G., Eumkeb G. (2018). *Boesenbergia rotunda* (L.) Mansf. extract potentiates the antibacterial activity of some β-lactams against β-lactam-resistant staphylococci. J. Glob. Antimicrob. Re..

[57] Ogawa T., Terao Y., Okuni H., Ninomiya K., Sakata H., Ikebe K., Maeda Y., Kawabata S. (2011). Biofilm formation or internalization into epithelial cells enable *Streptococcus pyogenes* to evade antibiotic eradication in patients with pharyngitis. Microb. Pathog..

[58] Post J.C., Stoodley P., Hall-Stoodley L., Ehrlich G.D. (2004). The role of biofilms in otolaryngologic infections. Curr. Opin. Otolaryngol. Head Neck Surg..

[59] Conley J., Olson M.E., Cook L.S., Ceri H., Phan V., Davies H.D. (2003). Biofilm formation by group a streptococci: Is there a relationship with treatment failure?. J. Clin. Microbiol..

[60] Hall-Stoodley L., Costerton J.W., Stoodley P. (2004). Bacterial biofilms: From the natural environment to infectious diseases. Nat. Rev. Microbiol..

[61] Rabin N., Zheng Y., Opoku-Temeng C., Du Y., Bonsu E., Sintim H.O. (2015). Biofilm formation mechanisms and targets for developing antibiofilm agents. Future Med. Chem..

[62] Manetti A.G., Zingaretti C., Falugi F., Capo S., Bombaci M., Bagnoli F., Gambellini G., Bensi G., Mora M., Edwards A.M. (2007). *Streptococcus pyogenes* pili promote pharyngeal cell adhesion and biofilm formation. Mol. Microbiol..

[63] Darsini D.T.P., Srinivasan P., Guna G., Manimekalai K., Dineshbabu J. (2015). In vitro anti-biofilm activity of *Piper longum* and *Piper nigrum* against clinical isolates of *S. pyogenes* isolated from pharyngitis patients. Int. J. Pharm..

[64] Mutalib L.Y., Nuraddin S.M., Aka S.T.H. (2015). Phytochemical screening, antibacterial and antibiofilm evaluation of *Lagenaria siceraria* fruit growing in Kurdistan region\Iraq. J. Pharmacogn Phytochem..

[65] Fuqua W.C., Winans S.C., Greenberg E.P. (1994). Quorum sensing in bacteria: The LuxR-LuxI family of cell density-responsive transcriptional regulators. J. Bacteriol..

[66] Clinical and Laboratory Standards Institute (2006). Methods for Dilution Antimicrobial Susceptibility Tests for Bacteria that Grow Aerobically: Approved Standard.

