# Supplementary figures and images for: Bactericidal and Anti-Biofilm Activity of Ethanol Extracts Derived from Selected Medicinal Plants against Streptococcus pyogenes
Source: Molecules. 2019 Mar 24;24(6):1165. doi: 10.3390/molecules24061165 (PMC6471238; doi:10.3390/molecules24061165)

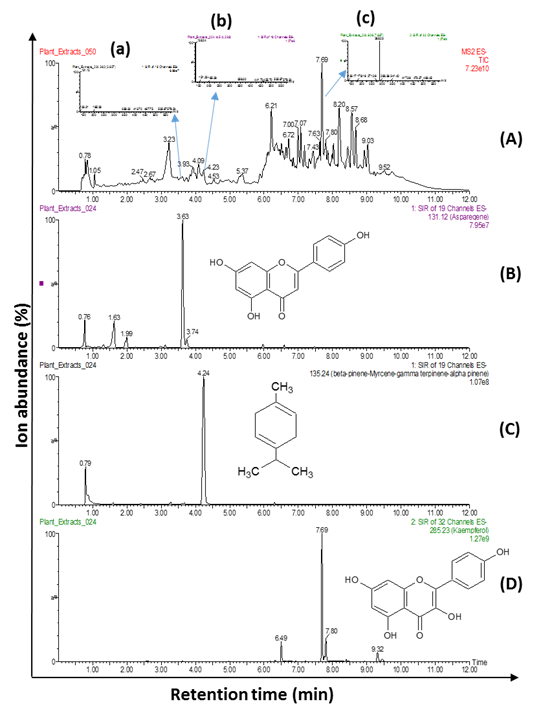

Supplement: Supplementary file 1 [file molecules-24-01165-s001.zip › molecules-455068-supplementary.png]
